# Bailout reconstruction of injured right internal thoracic artery in minimally invasive coronary artery bypass grafting

**DOI:** 10.1016/j.xjtc.2025.08.023

**Published:** 2025-09-12

**Authors:** Hiroki Moriuchi, Mamoru Orii, Takayuki Fujii, Nobuhiro Shimabukuro, Kohei Narayama, Akihiko Yamauchi

**Affiliations:** Department of Cardiovascular Surgery, Yuai Medical Center, Tomigusuku, Okinawa, Japan

**Figure:**
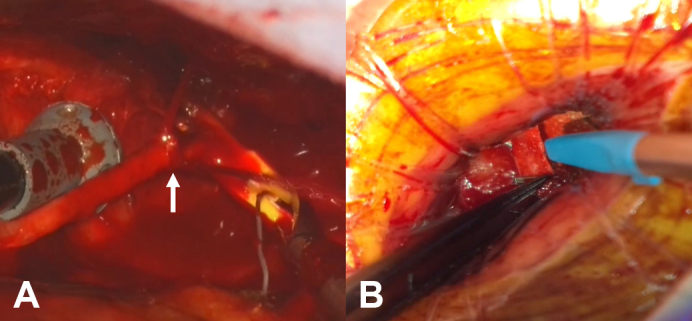
A, RITA injured during harvest. The *white arrow* indicates bleeding from the injured RITA. B, Good surgical view via second ICS.

**Central Message**

A right second intercostal approach allows safe bailout reconstruction of injured right internal thoracic artery using a great saphenous vein graft in minimally invasive coronary artery bypass grafting, avoiding sternotomy and preserving planned graft strategy.

Minimally invasive coronary artery bypass grafting (MICS CABG) is being increasingly performed owing to its less invasive nature. However, bilateral internal thoracic artery (BITA) harvesting in MICS CABG requires technical challenges, especially in patients with tight adhesions.^1^ We present a bailout technique in a case of right internal thoracic artery (RITA) injury during MICS CABG, successfully managed by graft extension with a great saphenous vein (GSV) via a right second intercostal space approach. Institutional Review Board approval was not required, and informed consent for publication was obtained from the patients.

## Case Description

**Figure 1:**
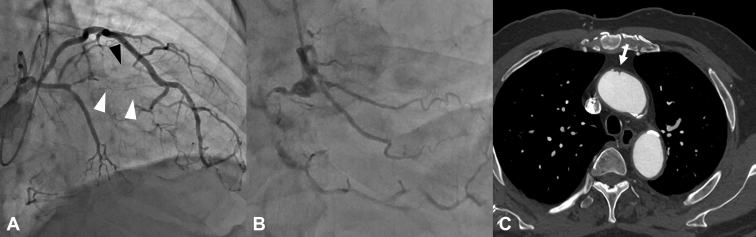
A, Coronary angiography demonstrated total occlusion of the left anterior descending artery (*white arrowhead*) and the diagonal branch (*black arrowhead*). B, Severe stenosis was observed at the ostium of the right coronary artery. C, Computed tomography demonstrated atheromatous changes in the ascending aorta (*white arrow*).

A 78-year-old man presented with chronic total occlusion of the left anterior descending artery and the diagonal branch, as well as 99% stenosis of the proximal right coronary artery (Figure 1). In the presence of severe ascending aortic calcification, MICS CABG with BITA was performed using an aorta nontouch strategy.

Under general anesthesia and intubation with a double-lumen tube, the patient was placed in a right semidecubitus position. A 10-cm left thoracotomy was performed through the fifth intercostal space, and we began harvesting the RITA in a skeletonized fashion using a harmonic scalpel (Ethicon Endo-Surgery). Severe adhesions between the RITA and the chest wall were noted, making harvesting extremely difficult. As a result, the RITA was injured at its proximal one-third segment. Because of substantial bleeding, hemostasis was achieved by clipping the RITA on both sides of the injured segment and dividing it. After the RITA injury, we immediately administered heparin (100 U/kg), then clamped and divided the RITA. A 5-cm incision was made in the right second anterior intercostal space, and the proximal portion of the divided RITA was carefully inspected through this approach. The RITA was then anastomosed to the great saphenous vein (GSV) at this site to extend its length and create an I-composite graft (Video 1). The composite conduit was used to perform sequential anastomoses to the diagonal branch and posterior descending artery.

**Figure 2:**
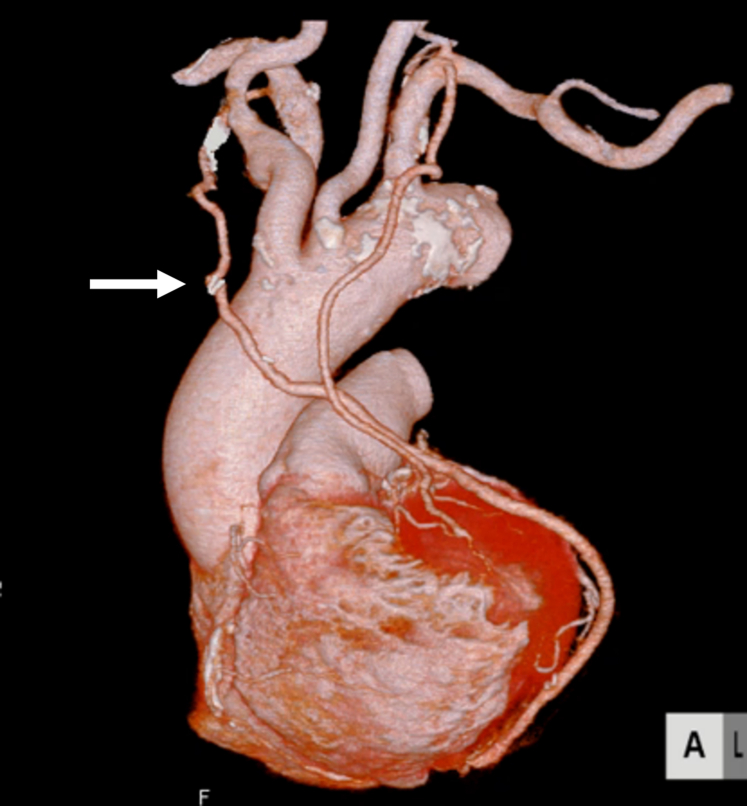
Postoperative computed tomography revealed all graft patency. The *arrow* indicates the anastomotic site between the right internal thoracic artery and the great saphenous vein.

Following anastomosis of the left internal thoracic artery (LITA) to the left anterior descending artery, good graft flow was confirmed, and the procedure was completed. Postoperative contrast-enhanced computed tomography (CT) confirmed graft patency and adequate positioning of the composite graft (Figure 2). The patient was discharged home without complications. Follow-up at 6 months demonstrated that the graft remained patent.

## Discussion

RITA harvesting in MICS CABG can be technically demanding due to limited access and anatomic variability. Adhesions from prior inflammation or anatomic constraints may lead to injury during harvesting. In such cases, quick and safe bailout is very important. In the event of ITA injury during MICS CABG, it may be necessary to revise the graft design, reduce the number of target vessels, or convert to median sternotomy.^2^ In our case, the right anterior second intercostal approach enabled proximal RITA–GSV anastomosis in a well-exposed view. This bailout technique successfully avoided median sternotomy while preserving the original graft design and maintaining all planned target vessel anastomoses. Sakai and colleagues^3^ reported that this approach enables access to the ascending aorta for proximal anastomosis in MICS CABG. We applied this technique to manage an intraoperative ITA injury. This approach provides excellent access to the injured RITA and allows safe and efficient reconstruction without extending the main thoracotomy. There are several alternative bailout strategies, including anastomosing a free RITA to the aorta, using a free RITA in a composite Y-graft configuration off the LITA, or substituting the RITA with a saphenous vein graft.

In our case, however, bleeding from the RITA was controlled promptly, and a satisfactory surgical view was obtained through the right second intercostal approach. This allowed us to extend the RITA with a saphenous vein graft without technical difficulty, and conversion to sternotomy was not required.

When planning MICS CABG with BITA use, preoperative imaging should be carefully evaluated. Because the ITA graft has a critical role in long-term survival, appropriate bailout options in the event of intraoperative complications should be prepared.

## Conflict of Interest Statement

The authors reported no conflicts of interest.

The *Journal* policy requires editors and reviewers to disclose conflicts of interest and to decline handling or reviewing manuscripts for which they may have a conflict of interest. The editors and reviewers of this article have no conflicts of interest.

## Supplementary Data

Video 1 The injured right internal thoracic artery was extended using a great saphenous vein via the right second intercostal space approach. Video available at: https://www.jtcvs.org/article/S2666-2507(25)00363-3/fulltext.
