# Supplementary figures and images for: Bailout reconstruction of injured right internal thoracic artery in minimally invasive coronary artery bypass grafting
Source: JTCVS Tech. 2025 Sep 12;34:123–5. doi: 10.1016/j.xjtc.2025.08.023 (PMC12683061; doi:10.1016/j.xjtc.2025.08.023)

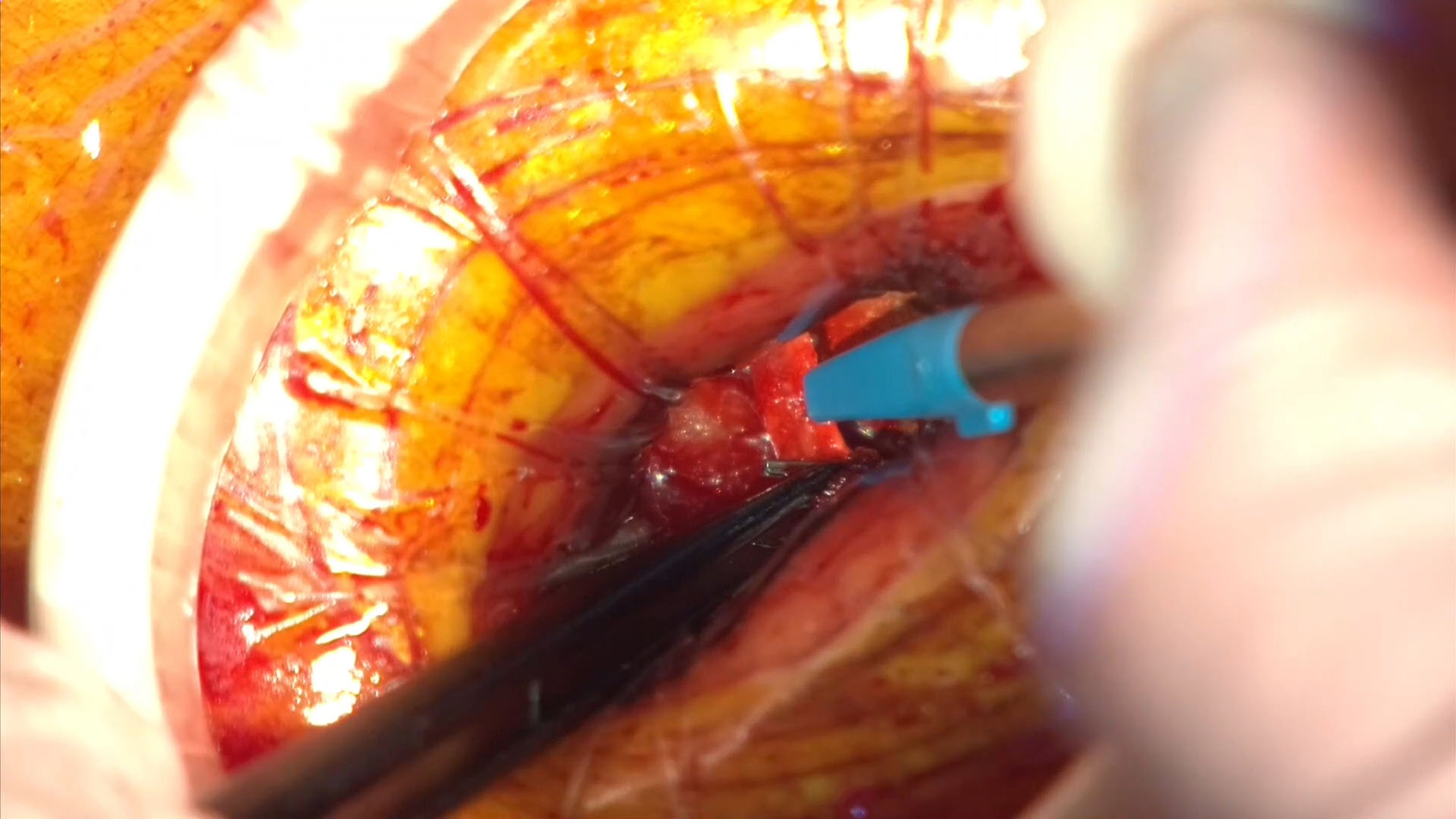

Supplement: Video 1 — The injured right internal thoracic artery was extended using a great saphenous vein via the right second intercostal space approach. Video available at: https://www.jtcvs.org/article/S2666-2507(25)00363-3/fulltext. [file fx2.jpg]
